# Review of recent evidence on the management of heartburn in pregnant and breastfeeding women

**DOI:** 10.1186/s12876-022-02287-w

**Published:** 2022-05-04

**Authors:** Raja Affendi Raja Ali, Jamiyah Hassan, Laurence J. Egan

**Affiliations:** 1 Gastroenterology Unit, Department of Medicine, Faculty of Medicine, Universiti Kebangsaan Malaysia Medical Centre, Kuala Lumpur, Malaysia; 2 GUT Research Group, Faculty of Medicine, Universiti Kebangsaan Malaysia, Kuala Lumpur, Malaysia; 3 Faculty of Medicine and Hospital, Universiti Teknologi MARA, Sungai Buloh, Selangor Malaysia; 4 Department of Clinical Pharmacology, Galway University Hospital, The National University of Ireland, Galway, Ireland

**Keywords:** Gastroesophageal reflux disease, Heartburn, Pregnancy, Breastfeeding, Treatment

## Abstract

Gastroesophageal reflux disease (GERD) is one the most common medical complaints in pregnant women. Some women continue to experience GERD symptoms after delivery. Effective management of GERD symptoms is important to improve productivity and quality of life. Management of heartburn in pregnant and breastfeeding women involves lifestyle modifications, dietary modifications, non-pharmaceutical remedies and pharmaceutical drugs. For most patients, lifestyle/dietary modifications are helpful in reducing GERD symptoms. For patients who require a more intense intervention, various types of pharmaceutical drugs are available. However, the suitability of each treatment for use during pregnancy and lactation must be taken into consideration. This article explores the reported efficacy and safety of these treatment options in pregnant and breastfeeding women. Recommended treatment algorithm in pregnant and breastfeeding women have been developed.

## Background

Gastroesophageal reflux disease (GERD) is one the most common medical complaints in pregnant women. Its prevalence has been reported to reach as high as 80% in certain populations [1–3]. The prevalence of GERD is also increased as pregnancy progresses from the first to third trimester [4, 5].

Regurgitation, acid taste in mouth and heartburn are among the most common GERD symptoms, with heartburn and regurgitation causing the most significant negative impact [1, 2, 6, 7]. Heartburn during pregnancy may be caused by hormonal changes which affects normal gastric motility, increased intra-abdominal pressure from the growing uterus, slower gastrointestinal transit time or weight gain as pregnancy progresses, leading to acid reflux [8–14]. Heartburn and acid reflux have also been shown to be associated with severity of nausea and vomiting during pregnancy [15].

GERD, especially nocturnal GERD, can have a negative impact on productivity and health-related quality of life [1, 6, 16, 17]. Emotion, sleep, eating/drinking, and physical/social functioning are all significantly affected by GERD, although the most significant impact is on sleep [17]. Effective management of GERD symptoms is important to improve quality of life. Treatment is aimed towards alleviating the symptoms caused by the acid reflux. Some guidelines suggest diet/lifestyle modifications and the use of medications to treat GERD symptoms [8, 18]. These include pharmacological agents such as antacids or alkali mixtures, H_2_ receptor antagonists (H_2_RA) or proton pump inhibitors (PPI) [8]. However, evidence-based recommendations on use of pharmacological agents during pregnancy and lactation have been lacking [9, 10].

Although GERD symptoms typically resolve following delivery, about 20% of women continue to experience GERD symptoms even after giving birth [19]. As these women may still require medications, it is important to consider the excretion of the medications in breast milk and its potential effect to the nursing infant.

This review aims to assess the efficacy and safety of various interventions for relieving heartburn during pregnancy and lactation, based on recent evidence.

## Methodology

An electronic search through online databases was conducted for relevant articles published in English between 2009 and 2020. The keywords used for the search engine to obtain relevant papers were: GERD, pregnancy, breastfeeding, lactation, treatment, antacid, alginate, proton pump inhibitor, PPI, histamine-2 receptor antagonist, H_2_RA, mucosal protectant, potassium-competitive acid blocker, P-CAB, promotility, prokinetic. Potential articles of interest were read through and then selected for inclusion in this review. Bibliographies of relevant articles were also checked to obtain additional articles. Studies of interventions in pregnant and breastfeeding females with GERD symptoms, dyspepsia, reflux, epigastric pain, and hyperemesis gravidarum, were included in this review.

## Review outcomes

### Non-drug interventions for heartburn relief during pregnancy and lactation

Women with symptoms of heartburn during pregnancy and lactation should first be advised on lifestyle and diet modifications (Table 1) [18]. For women with mild symptoms, a non-pharmacological approach may be all that is required to alleviate symptoms.

**Table 1:** Recommended lifestyle, dietary and medicine intake modifications for heartburn relief in pregnant and breastfeeding women

Lifestyle modifications
Avoid eating within 3 h of going to bed [19, 68] Elevate the head of bed by 10–15 cm [19, 69] Lie down on the left side, rather than the right side or supine [24, 70] Avoid tobacco use [19, 71] Weight loss is recommended for overweight breastfeeding mothers [28, 72] Maintain an upright posture, especially after eating [8] Chew gum to neutralise acid [12, 73] Increase physical activity to help with gastric motility [12, 14]
Dietary changes
Abstain from alcohol intake [19, 72, 74] Avoid trigger foods and beverages (e.g. fatty or spicy foods, chocolate, mints, caffeinated beverages, citrus juices, tomatoes and carbonated products) [8, 19, 72, 75–77] Consume frequent small meals [10, 14] Drink fluids between meals, and limit fluid intake with meals [7, 12, 14] Keep a food diary to identify trigger foods [12, 14]
Medicinal intake modifications
Avoid medications that decrease LOS pressure [24] Avoid potentially harmful medications (e.g. anticholinergics, calcium channel antagonists, theophylline, antipsychotic agents, antidepressants) [24]

*LOS* lower oesophageal sphincter

### Non-pharmaceutical management of GERD during pregnancy

Ginger (*Zingiber officinale*) has frequently been used to treat indigestion, nausea and vomiting. A review on the effects of ginger in pregnancy-induced nausea and vomiting has established ginger as a safe alternative for accelerating gastric emptying and improving nausea and vomiting during pregnancy, although additional controlled studies are needed to confirm these hypotheses [20].

In a prospective hospital-based study, 64 pregnant women in the first trimester were interviewed for symptoms of GERD and dietary details, and followed up until term and delivery. Consumption of green vegetables were found protective against heartburn (relative risk, RR 15; 95% confidence interval, CI 3.52–63.89) and more frequent consumption proportionally decreased the risk of GERD (trend χ^2^ < 0.001) [21].

A two-phase, randomised, active-controlled, open-label, parallel-group clinical trial was carried out to compare the efficacy of quince fruit extract against ranitidine in 137 pregnant women with GERD symptoms. Ranitidine was prescribed at 150 mg twice daily and quince fruit extract was taken at 10 mg after meals, for a duration of 4 weeks. After 2 weeks, pregnant women taking the quince extract had significantly lower mean General Symptom Score (GSS) score than those receiving ranitidine (*p* = 0.036). However, the GSS values after 4 weeks were not significantly different between groups (*p* = 0.074); quince extract was reported to have similar efficacy to ranitidine in the management of GERD during pregnancy [22].

A study reported that pregnant women in Jordan relieve heartburn with remedies such as cold milk, a ‘baking powder’ liquid, herbs, cucumber, lintel seeds, or dry tea leaves. The study participants considered their chosen remedy to be "useful". However, although these strategies may eliminate the need for medications, it cannot be considered non-toxic. Thus, home remedies without scientific basis are preferably avoided during pregnancy [23].

### Pharmaceutical management of GERD during pregnancy and lactation

For most patients, lifestyle/dietary modifications are helpful, but may not be entirely sufficient in controlling GERD symptoms. A step-up program with increasing intensification of treatment is commonly utilised for patients with persistent GERD symptoms. The optimum management of GERD in pregnant and breastfeeding women requires consideration of the drugs' safety as well as efficacy, since the medications may affect the foetus/infant [24]. The effect of specific medications on the foetus/infant during pregnancy and lactation are often not well understood, due to ethical limitations against involving pregnant/breastfeeding women in clinical drug trials. Even so, based on animal studies and/or population-based studies involving pregnant women, certain medications appear to be safe for use in pregnancy. These medications may be a helpful alternative if lifestyle modifications do not adequately relieve symptoms [14].

GERD during pregnancy is known to predispose patients to postpartum GERD [25–27]. This may necessitate the need to continue GERD symptom management even after delivery (into the breastfeeding period). However, data on the use of medications for GERD while breastfeeding are limited [28]. Medications with minimal systemic absorption are preferred for breastfeeding patients [28].

#### Antacids

Antacids are commonly used for the treatment of GERD during pregnancy. Its key ingredient consists of various salts of calcium, magnesium and aluminium. Its mechanism of action is by neutralising the stomach acid and by inhibiting pepsin [29]. Antacids are considered as non-systemic therapy, and thus are a favourable first line therapy to manage GERD during pregnancy [12].

However, because gastric acidity is essential for the absorption of certain minerals (e.g. calcium, iron, magnesium) and vitamin B12, antacids should be used with caution in pregnant patients with deficiencies [11, 24, 30]. Antacids should also not be taken within two hours of iron and folic acid supplements [31].

Antacids containing aluminium salts are considered safe for use in pregnant women. Long-term intake of antacids containing magnesium trisilicate have been associated with cardiovascular problems, respiratory issues, hypotonia, and kidney stone formation. Sodium bicarbonate antacids are preferably avoided during pregnancy, as they may lead to fluid overload and metabolic alkalosis in the mother and foetus [12].

Excessive use of calcium-containing antacids (> 1000 mg elemental calcium/day) is to be avoided in pregnant women, as calcium may cross the placenta [29, 32]. It may also lead to calcium-alkali syndrome, characterised by hypercalcaemia, metabolic alkalosis, and renal impairment [32, 33]. Dehydration due to vomiting can make hypercalcemia and alkalosis potentially life-threatening [32, 33]. Events involving hypercalcaemia may occur with self-administration of over-the-counter medications. Pregnant women with GERD symptoms must receive guidance to prevent potential overdose [34, 35].

Data on use of antacids when breastfeeding are lacking, although antacid use during breastfeeding is considered generally acceptable. Aluminium, calcium, or magnesium are poorly absorbed orally. Furthermore, magnesium and calcium are usual constituents of breast milk, and breast milk has lower levels of aluminium than cow’s milk and infant formula [29, 36, 37].

#### Alginates

**Fig. 1:**
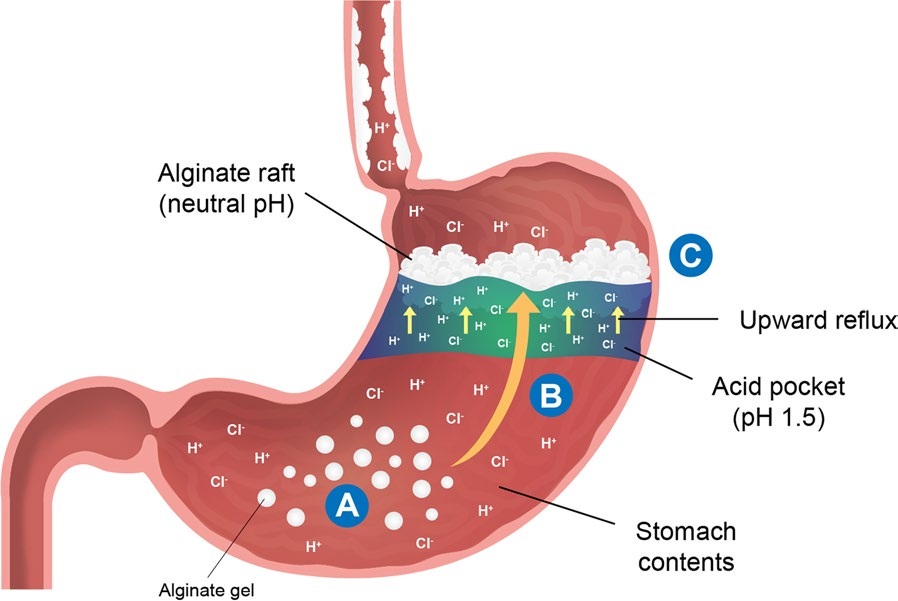
Diagram of alginate raft structure formation following ingestion of an alginate-containing formulation. Alginates react with the stomach acid to form a gel-like substance with neutral pH (**A**). Sodium bicarbonate contained in the formulation releases carbon dioxide gas, which becomes trapped in the gel. The trapped carbon dioxide gas allows the gel to float to the surface of the stomach content (**B**), effectively forming a raft structure (**C**) which acts as a barrier over the gastric acid pocket, and blocks against upward reflux. Adapted from Bor et al. [79]

Alginates are natural polysaccharide polymers which polymerise into a gel upon contact with gastric acid [38]. Alginate formulations for GERD treatment are frequently combined with antacids such as sodium bicarbonate. The sodium bicarbonate component in the alginate formulation releases carbon dioxide within the alginate gel; this causes the gel to float to the top of the stomach contents to form a raft structure [38]. The alginate-antacid raft forms a non-systemic barrier over the postprandial acid pocket to reduce postprandial acid reflux (Fig. 1) [38]. In an alginate/antacid combination, the rapid antacid action works concurrently with the long-lasting alginate reflux suppression [39].

A double-blinded, randomised, controlled trial to compare the efficacy of an alginate-based reflux suppressant, Liquid Gaviscon® (Reckitt Benckiser Healthcare (UK) Ltd, Hull, UK; Liquid Gaviscon® contains 500 mg sodium alginate, 267 mg sodium bicarbonate and 160 mg calcium carbonate per 10 mL) vs. a magnesium–aluminium antacid gel (Maalox® Olic (Thailand) Co., Ltd., Thailand; 5 mL contains 120 mg magnesium hydroxide and 220 mg aluminium hydroxide) in 100 pregnant women at < 36 weeks' gestation who had ≥ 2 episodes of heartburn a week. The treatment dose was 15 mL orally three times after meal and before bedtime, continued for 2 weeks. Both treatments similarly improved heartburn frequency (80% vs. 88%, *p* = 0.275) and heartburn intensity (92% vs. 92%, *p* = 1.000). Alginate-based reflux suppressant was shown no different from magnesium–aluminium antacid gel in the treatment of heartburn in pregnancy [40].

A multicentre, prospective, open-label study to evaluate the efficacy and safety of Liquid Gaviscon® in pregnant women (≤ 38 weeks' gestation) with heartburn and/or reflux was conducted. Treatment was at a dose of 10–20 mL as required to relieve symptoms, to a maximum of 80 mL per day, for 4 weeks. The investigators considered the treatment a success in 91% of patients (95% CI 85.0–95.3), and 90% (95% CI 84.1–94.8) when self-assessed by the patient. Adverse events were very few in this study. Liquid Gaviscon® was shown to not impact the serum sodium levels during pregnancy; this is important as hypertension, pre-eclampsia, and oedema are all significant complications during pregnancy [26].

An open-label, multicentre, phase IV study investigated the efficacy and safety of an alginate-based reflux suppressant containing potassium bicarbonate (Gaviscon Advance®; Reckitt Benckiser Healthcare (UK) Ltd, Hull, UK) with a significantly lower sodium content per dose vs. conventional alginate formulations in the treatment of heartburn during pregnancy. A total of 150 pregnant women before or at 38 weeks' gestation took 5–10 mL of Gaviscon Advance® (up to 40 mL/day), as required, for 4 weeks. At the end of the study, the investigators and women rated the efficacy of treatment as "very good"/"good" in 88% and 90% of women, respectively. Over half of the women experienced symptom relief within 10 min. Most adverse events reported by women in this study were considered related to the pregnancy, and not related to the study medication. In addition, the incidence of adverse events affecting the foetus/baby was low and consistent with expected incidences, and none was considered related to the study medication. The maternal mean sodium or potassium serum concentrations saw minimal change after 4 weeks of treatment [41].

Maternal alginate absorption is limited and alginates are not significantly metabolised. Thus, alginates are considered acceptable for use during lactation [36, 37]. Its mode of action and long-term experience with its use indicate that they are safe to use in high-risk pregnancies and breastfeeding populations [26].

#### Histamine-2 receptor antagonist (H_2_RA)

H_2_RAs are the most commonly used medication in pregnant women for the treatment of GERD symptoms which failed to respond to antacids. H_2_RAs act by competitively inhibiting histamine at H_2_ receptors of the parietal cells in the stomach, which results in gastric acid secretion inhibition [19, 42]. H_2_RAs which have previously been used for GERD symptom alleviation during pregnancy include cimetidine, ranitidine, famotidine and nizatidine [19].

A meta-analysis performed to determine the foetal safety of H_2_RA use during pregnancy compared 2398 pregnant women who received H_2_RAs in at least the first trimester against 119,892 women in the control group. The meta-analysis revealed no increased risk for congenital malformation, spontaneous abortion, preterm birth, and small for gestational age infants vs. the control group [43].

Different H_2_RAs are excreted in breast milk at lower amounts than the doses administered to infants. H_2_RAs are also known to stimulate prolactin secretion, although the impact of such a secretion in nursing mothers is not known [36].

Cimetidine is excreted into breast milk in largest amounts among H_2_RAs, with a dose-normalised peak level at 2.5–3.1 mg/L for each 100 mg of cimetidine [36, 44]. Ranitidine and nizatidine are both excreted in small amounts in milk; ranitidine concentration peaks at 2.6 mg/L 5.5 h after a 150 mg dose, whereas nizatidine concentration peaks at 1.2 mg/L about 2 h after a 150 mg dose [36, 45, 46]. However, animal toxicology studies have suggested that nizatidine may be unsuitable for use during breastfeeding [36]. It is important to note that the US FDA has issued statement to withdraw all ranitidine products from the market, due to the possible higher-than-safe levels of a carcinogenic contaminant, NDMA, in ranitidine products [47].@@@ Famotidine, on the other hand, is least excreted into breast milk (concentration in breast milk at 53 and 55 µg/L at 3 and 6 h after a 10/20 mg dose, respectively), and is the longest acting between the H_2_RAs [48]. Famotidine may be the preferred H_2_RA to use during lactation [36].

#### Mucosal protectant

Sucralfate is a mucosal protectant which adheres to the epithelial cells, creating a physical barrier against the acidic environment [42, 49]. When taken at 1 g thrice daily, it has been shown effective in managing GERD symptoms without adverse maternal or foetal events [12]. As sucralfate is minimally-absorbed, it is considered safe for use in pregnancy [37].

Sucralfate use during lactation has not been studied, although it is likely safe due to limited maternal absorption [24].

#### PPI

In the acidic environment, PPIs irreversibly bind to the H^+^/K^+^-ATPase to prevent gastric acid production [19, 50]. However, PPI-induced gastric acid inhibition has a delayed onset [19, 51]. Many patients with GERD symptoms report dissatisfaction with PPI treatment [50, 51].

Although most PPIs have been classified as category B drug in pregnancy, PPI use in pregnancy is reserved to those with GERD complications or those with symptoms not responding to other therapies [12]. Omeprazole is classified as a category C drug, due to risk shown in animal studies [12].

Although there are potential adverse effects which have been associated with long-term PPI use includes kidney disease, dementia, bone fracture, myocardial infarction, infections, micronutrient deficiencies, and gastrointestinal malignancies, the strength of evidence connecting these adverse effects to PPIs is low; therefore, the connection between long-term PPI use with these possible adverse events are inconclusive [30]. However, PPI therapy causes increased gastric pH, which may deplete the protective barrier function against ingested pathogens and increase vulnerability to enteric infections [7, 52].

In a cohort study involving 6051 nulliparous women, PPI use anytime during pregnancy increased the risk of overall preeclampsia (adjusted odds ratio, aOR 1.17; 95% CI 1.04–1.32) and preeclampsia at term (aOR 1.20; 95% CI 1.04–1.39). However, use of PPI after 28 gestational weeks reduced the risk of preterm (delivery < 37 weeks) preeclampsia (aOR 0.63; 95% CI 0.41–0.96) and early (delivery < 34 weeks) preeclampsia (aOR 0.41; 95% CI 0.20–0.82). The authors report that the findings may be due to heterogeneity of preeclampsia, with PPIs potentially preventing preterm preeclampsia when taken near preeclampsia onset [53].

In a cohort study involving 840,968 live births to study the association between PPI use during pregnancy and the risk of major birth defects, 5082 births were determined to have PPI exposure between 4 weeks prior to conception and the end of the first trimester. Major birth defects were reported in 3.4% of infants of exposed mothers, compared to 2.6% of infants of unexposed mothers (adjusted prevalence odds ratio: 1.23; 95% CI 1.05–1.44). Among 3651 infants with exposure limited to the first trimester, major birth defects were reported in 3.2% of the infants (adjusted prevalence odds ratio: 1.10; 95% CI 0.91–1.34). Lansoprazole intake within 4 weeks prior to conception was significantly associated with an increased risk of birth defects. However, the risk of birth defects was not significantly increased with individual PPI use during the first trimester. Interestingly, omeprazole was the most commonly prescribed PPI in this study [54].

A meta-analysis of 7 studies reported no significant risk of birth defects with PPI use in the first trimester. Using data from 1530 PPI-exposed and 133,410 PPI-unexposed subjects, the overall OR for major malformations was 1.12 (95% CI 0.86–1.45). No increased risk for spontaneous abortions (OR 1.29; 95% CI 0.84–1.97) and preterm delivery (OR 1.13; 95% CI 0.96–1.33) were observed. A secondary analysis of 1341 omeprazole-exposed and 120,137 omeprazole-unexposed subjects revealed an OR for major malformations of 1.17 (95% CI 0.90–1.53). The results suggested that PPIs are not associated with an increased risk of major congenital birth defects, spontaneous abortions, or preterm births [55].

PPI excretion into breast milk is minimal; furthermore, stomach acid degrades PPIs. Therefore, PPIs may be broken down in the infant’s stomach. In addition, similar to H_2_RAs, PPIs may also raise serum prolactin [36].

In a study involving 12 mothers taking pantoprazole, pantoprazole was undetectable in the breast milk approximately 80% of the time. The concentration of pantoprazole in breast milk after 7 days of therapy was only 150 ng/L on average [56]. A nursing woman who ingested of 40 mg of pantoprazole was observed to excrete pantoprazole in her breast milk. The infant's intake of pantoprazole through breast milk was estimated at 7.3 µg, or 0.14% of the weight-normalised dose received by the mother. However, the infant was expected to absorb even lower amounts than this, as the pantoprazole would have been exposed to the infant's stomach acid [57].

Pantoprazole and omeprazole are both excreted into breast milk at 300–600 times lower amounts than doses given to infants [36]. This may be true for esomeprazole as well, being the single-isomer form of omeprazole. However, esomeprazole strontium is not preferable, as strontium is absorbed into the bone [36].

Data on lansoprazole use during breastfeeding is limited. As lansoprazole is safe for use in infants, it can be postulated that the amount of lansoprazole excreted in breast milk may be safe. Furthermore, the R-enantiomer of lansoprazole, dexlansoprazole, is assumed to also be safe [37].

The effect of rabeprazole use during breastfeeding is not available. Alternate drugs are recommended when nursing infants [37].

#### Potassium-competitive acid blockers (P-CAB)

P-CABs reversibly inhibit gastric H^+^/K^+^-ATPase by competitive interaction with the K^+^ site of the enzyme. P-CABs are absorbed rapidly and reach high plasma concentrations, and have a faster onset of action than conventional PPIs [42, 50]. However, in some classes of P-CABs, the binding to H^+^/K^+^-ATPase is quickly reversed, which may cause reduced efficacy [51].

The P-CAB known as vonoprazan has been in approved in Japan for erosive oesophagitis, gastric and duodenal ulcer, GERD, and *Helicobacter pylori* eradication [51]. However, to date, there is yet a clinical study conducted to evaluate the use of vonoprazan in pregnant women [58]. In a rat toxicology study, embryo-foetal toxicity was observed following exposure of > 28 times the maximum clinical dose (40 mg/day) of vonoprazan. Vonoprazan should not be prescribed to pregnant women, unless the expected therapeutic benefit is thought to outweigh any possible risk [58].

It is unknown whether vonoprazan is excreted in breast milk, although animal studies have shown that vonoprazan was excreted in milk. Breastfeeding should be avoided if taking vonoprazan [58].

#### Promotility drugs

Promotility drugs enhances gastric emptying, which reduces the time that the acid pocket sits in the stomach. Metoclopramide is one such promotility agent; it promotes gastrointestinal motility and gastric emptying, and increases LOS pressure [12, 59].

A retrospective cohort study analysed the safety of metoclopramide use during the first trimester of pregnancy in 3458 exposed infants. Metoclopramide was found not associated with adverse outcomes, which include major congenital malformations, low birth weight, preterm birth, and perinatal death [60].

The amount of metoclopramide excreted in breast milk may vary. Most infants will receive < 10% of the maternal weight-adjusted doses, although some infants may receive pharmacologically active doses [37]. No side effect was observed in infants whose mothers take 45 mg/day [28]. No adverse effects have been reported in breastfed infants with metoclopramide [37].

It is important to note the US FDA has issued a black box warning for metoclopramide due to reports of tardive dyskinesia with high-dose, long-term use [61]. Metoclopramide has also been shown to increase QT/RR slope and QT variance, thus caution is needed if considering for long-term use [62].

### Association of prenatal exposure to acid suppressors with childhood asthma and allergic disease

Several studies have shown that exposure to acid suppressors during gestation may be associated to childhood asthma, although maternal asthma was potentially a main confounder [63].

Mulder et al. identified three studies which reported an association between prenatal exposure to acid-suppressive drugs (ASDs) and childhood asthma. ASDs exposure increased the odds of asthma in toddlers by 85% (OR 1.85; 95% CI 1.07–3.19). Exposure to over 14 defined daily doses of any ASD increased the odds of asthma in toddlers by 156% (OR 2.56; 95% CI 1.18–5.52) [64].

A study involving 685,015 singleton children born between 1999 and 2007 in Sweden was carried out to investigate the relationship between maternal use of various drug types and the risk of childhood asthma. Drugs for GERD may be associated with childhood asthma (aOR 1.32; 95% CI 1.18–1.54), although further studies are required to verify the association [65].

A systematic review and meta-analysis associated ASD exposure during pregnancy with the risk of childhood asthma (RR 1.45; 95% CI 1.35–1.56; I^2^ = 0%; *p* < 0.00001). The overall risk of childhood asthma was increased with PPI use (RR 1.34; 95% CI 1.18–1.52; I^2^ = 46%; *p* < 0.00001) and H_2_RA use (RR 1.57; 95% CI 1.46–1.69; I^2^ = 0%; *p* < 0.00001). However, maternal asthma may be a main confounder in the selected studies. In addition, the adjusted hazard ratios (aHRs) for pre-existing GERD and GERD diagnosed during pregnancy were 1.07 (95% CI 0.79–1.44) and 1.17 (95% CI 0.92–1.48), respectively. This possibly supports GERD as a risk factor for childhood asthma. The study recommends caution when interpreting the link between prenatal exposure to ASDs and childhood asthma [66].

In a cohort study involving 33 536 children followed-up for a maximum of 8 years, the aHR for any allergic disease in children exposed to PPIs or H_2_RAs was 1.37 (95% CI 1.14–1.66). PPIs and/or H_2_RAs exposure during pregnancy was associated with atopic dermatitis, asthma and allergic rhinitis (aHRs: 1.32 [95% CI 1.06–1.64], 1.57 [95% CI 1.20–2.05] and 2.40 [95% CI 1.42–4.04], respectively). The risk for the development of ≥ 2 and 3 allergic diseases were also increased (aHRs: 2.13 [95% CI 1.43–3.19] and 5.18 [95% CI 2.16–12.42], respectively). PPI and H_2_RA intake during pregnancy may lead to an increased risk of atopic dermatitis, asthma and allergic rhinitis, and the development of multiple allergic diseases [67].

### Recommended treatment algorithm for pregnant and breastfeeding women

**Fig. 2:**
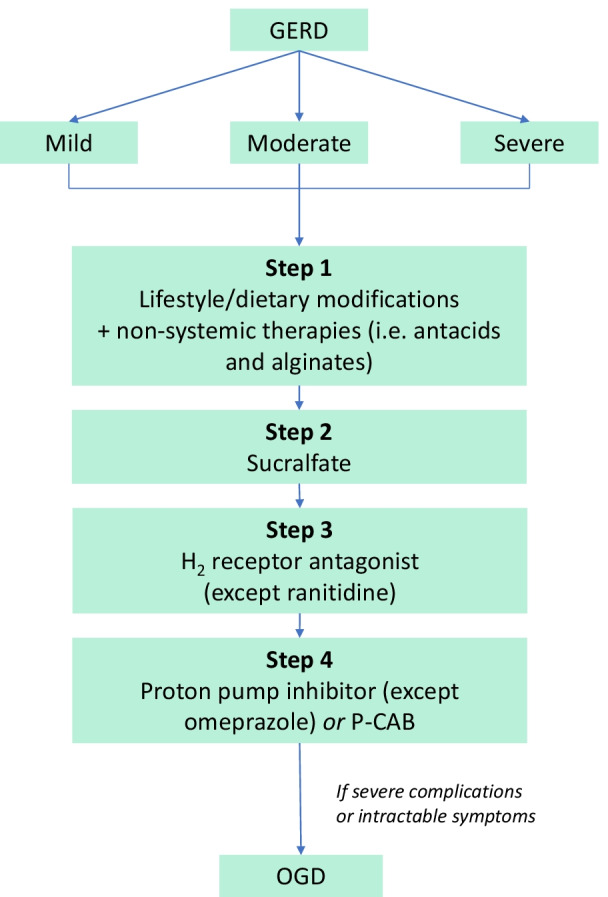
Preferred treatment algorithm for GERD during pregnancy, according to current evidence. Mild GERD is usually remedied by non-pharmaceutical interventions (step 1). In all patients, if symptoms persist after step 1, patients may proceed to step 2, and so on. Once symptoms are resolved, advise patients to continue non-pharmaceutical intervention. Adapted from Ali and Egan [24]. *GERD* gastroesophageal reflux disease, *OGD* oesophagogastroduodenoscopy, *P-CAB* potassium-competitive acid blockers

**Fig. 3:**
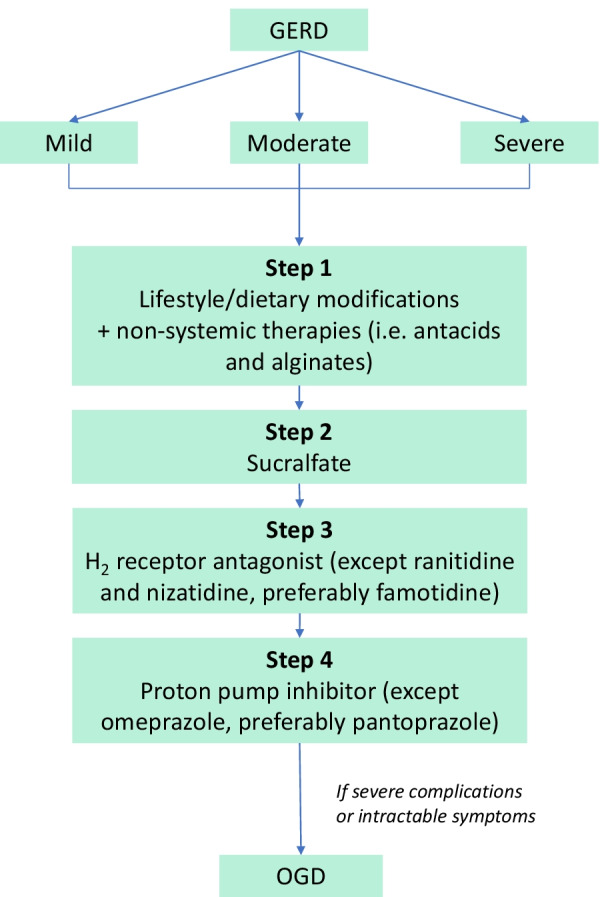
Preferred treatment algorithm for GERD during lactation, according to current evidence. Mild GERD is usually remedied by non-pharmaceutical interventions (step 1). In all patients, if symptoms persist after step 1, patients may proceed to step 2, and so on. Once symptoms are resolved, advise patients to continue non-pharmaceutical intervention. Adapted from Ali and Egan [24]. *GERD* gastroesophageal reflux disease, *OGD* oesophagogastroduodenoscopy

The safety of various GERD therapies during pregnancy and lactation are summarised in Table 2. The recommended treatment algorithm to alleviate symptoms of GERD in pregnant and breastfeeding women are shown in Figs. 2 and 3, respectively.

**Table 2:** Safety of GERD therapies during pregnancy and lactation

Medications	US FDA classification according to foetal safety^a^ [12, 47, 67]	Pregnancy		Lactation	
		Safety	Comments	Safety	Comments
Antacids					
Aluminium hydroxide	B	Yes, except for magnesium trisilicates and sodium bicarbonate	Care must be taken for use in pregnant women with nutrient deficiency	Yes, except for magnesium trisilicates and sodium bicarbonate	Not concentrated in breast milk [57]
Magnesium hydroxide	B				
Calcium carbonate	C				
Magnesium trisilicates	None				
Sodium bicarbonate	C				
Alginates	None	Yes	Likely safe due to limited maternal absorption	Yes	Likely safe due to limited maternal absorption
H_2_RA					
Cimetidine	B	Yes, except ranitidine	Any H_2_RA may be used	Yes, except ranitidine	Famotidine is preferred
Ranitidine	B^b^				
Famotidine	B				
Nizatidine	B				
Mucosal protectant					
Sucralfate	B	Yes	Likely safe due to limited maternal absorption	Yes	Minimal excretion in breast milk [57]
PPI					
Omeprazole	C	Yes, except omeprazole	PPIs except omeprazole are considered appropriate if GERD is poorly controlled by other interventions	Yes, except omeprazole	Pantoprazole is preferred
Lansoprazole	B				
Rabeprazole	B				
Pantoprazole	B				
Esomeprazole	B				
P-CAB					
Vonoprazan	None	Unknown	–	Unknown	–
Promotility agents					
Metoclopramide^c^	B	No	Long-term use is not recommended [78]	No	Long-term use is not recommended [78]

US FDA, United States Food and Drug Administration; GERD, gastroesophageal reflux disease; H_2_RA, histamine-2 receptor antagonist; PPI, proton pump inhibitor; P-CAB, potassium-competitive acid blocker

^a^The US FDA classifies drugs according to foetal safety, as follows: Category A drugs as the safest category; Category B drugs are considered relatively safe; category C drugs are likely safe or negligibly harmful; category D drugs are potentially dangerous; and category X drugs are contraindicated during pregnancy

^b^The U.S. Food and Drug Administration has recently issued statement to request manufacturers withdraw all prescription and over-the-counter ranitidine drugs from the market, due to ongoing investigations on the possible higher-than-safe levels of a carcinogenic contaminant, *N*-nitrosodimethylamine (NDMA), in ranitidine products[47]

^c^The US FDA issued a black box warning for metoclopramide due to reports of tardive dyskinesia with high-dose, long-term use[61]

## Conclusions

Management of heartburn in pregnant and breastfeeding women involves lifestyle or dietary modifications, non-pharmaceutical remedies and pharmaceutical drugs. For most patients, lifestyle/dietary modifications are helpful in reducing GERD symptoms. Alginate/antacid combinations may be also considered for its capacity to develop a non-systemic mechanical barrier above the postprandial acid pocket in the stomach [38]. Patients who require more intense intervention may be offered pharmaceutical drugs. However, the suitability of each treatment for use during pregnancy and lactation must be taken into consideration.

## Data Availability

Not applicable.

## Abbreviations

aHR: Adjusted hazard ratios
aOR: Adjusted odds ratio
ASD: Acid-suppressive drug
BMI: Body mass index
GERD: Gastroesophageal reflux disease
GSS: General Symptom Score
H_2_RA: H_2_ receptor antagonist
LOS: Lower oesophageal sphincter
OR: Odds ratio
P-CAB: Potassium-competitive acid blocker
PPI: Proton pump inhibitors
RR: Relative risk
TLOSR: Transient lower oesophageal sphincter relaxation
